# Diagnostic Value of CK-18, FGF-21, and Related Biomarker Panel in Nonalcoholic Fatty Liver Disease: A Systematic Review and Meta-Analysis

**DOI:** 10.1155/2017/9729107

**Published:** 2017-02-23

**Authors:** Lei He, Linfeng Deng, Quan Zhang, Jianli Guo, Jinan Zhou, Wenjian Song, Fahu Yuan

**Affiliations:** ^1^Department of Blood Transfusion, Tongji Hospital, Tongji Medical College, Huazhong University of Science and Technology, Wuhan, China; ^2^Department of Laboratory Medicine, Hubei Provincial Hospital of Integrated Chinese & Western Medicine, Wuhan, China; ^3^Department of Biochemistry and Molecular Biology, School of Basic Medicine, Tongji Medical College, Huazhong University of Science and Technology, Wuhan, China; ^4^School of Medicine, Jianghan University, Wuhan, China

## Abstract

Liver biopsy still remains the gold standard for diagnosing nonalcoholic steatohepatitis (NASH), but with limitations. There is an urgent need to develop noninvasive tests that accurately distinguish NASH from simple steatosis. The purpose of this meta-analysis was to evaluate the diagnostic value of serum biomarkers including cytokeratin 18 (CK-18), fibroblast growth factor 21 (FGF-21), and combined biomarker panel (CBP) in the diagnosis of NAFLD, especially NASH. A total of 25 studies met the inclusion criteria. Pooled sensitivity and specificity values for chosen serum markers for diagnosing NASH are as follows: CK-18 (M30), 0.75 and 0.77; CK-18 (M65), 0.71 and 0.77; FGF-21, 0.62 and 0.78; and CBP, 0.92 and 0.85. CBP demonstrated better accuracy with higher sensitivity and specificity than those tested individually. Furthermore, the AUROC of CBP was 0.94 (95% CI, 0.92–0.96), compared to CK-18 or FGF-21 assay, which showed the most significant ability to distinguish NASH from simple steatosis. The results suggest that increased circulating CK-18 and FGF-21 are associated with NASH and may be used for initial assessment, but not enough. Importantly, CBP is potentially used as accurate diagnostic tools for NASH. Further prospective designed studies are warranted to confirm our findings.

## 1. Introduction

Nonalcoholic fatty liver disease (NAFLD) has become an important public health problem because of its high prevalence and potential progression to severe liver disease. Steatosis, the hallmark feature of NAFLD, refers to excessive amount of lipid in liver, and the processes are linked to lipid metabolism disorders [1–3]. In some individuals, steatosis progresses to nonalcoholic steatohepatitis (NASH), which encompasses spectrum of hepatic pathological changes including inflammation, apoptosis, and fibrosis. Above all, NASH is a potentially serious condition, because as many as 20% patients in whom may progress to cirrhosis or hepatocellular carcinoma (HCC) [4, 5].

Importantly, NAFLD/NASH as the hepatic entity of the metabolic syndrome may itself pose a risk factor for HCC, even in the absence of cirrhosis. As observed from Ertle et al. [6], patients with NAFLD/NASH-associated HCC exhibited a higher prevalence of metabolic features; 41.7% of them had no evidence of cirrhosis. Thus, the possibility of HCC derived from noncirrhotic NASH liver would be an even greater impetus for early detection, early prevention, and early treatment. At present, liver biopsy is the gold standard for diagnosing NAFLD. There is no doubt that the biopsy for this disease has several important limitations. Firstly, it is an invasive procedure that requires hospitalization. Furthermore, the procedure may result in major complications like hemorrhage and may not be acceptable by some patients. The relative complexity and high costs of the gold standard approach employed for diagnosing the vast majority of NAFLD have urged the field to search for alternative diagnostic methods.

Due to the important limitations of liver biopsy, lots of studies have tried to identify potential novel biomarkers based on the current knowledge of the pathophysiologic mechanisms involved in the progression of NAFLD. The available biomarkers should be simple, repeatable, inexpensive, and accurate for a particular disease process, specifically for distinguishing NASH from simple steatosis, predicting risk of disease progression [7]. Such a test would not only aid clinicians in the identification of patients with NASH, but also allow for noninvasive frequent monitoring of disease progression and response to therapy. Recently, one important review [8] summarized several novel circulating biomarkers including cytokeratin 18 (CK-18), fibroblast growth factor 21 (FGF-21), and related biomarker panel combining more than two kinds of serum proteins. These biomarkers are released into circulation as a consequence of oxidative stress, hepatocyte apoptosis, or inflammation in response to lipid metabolism disorders because of NAFLD. However, how to select the most accurate biomarker is still controversial. To the best of our knowledge, only a few studies focus on synthetic evaluation and identify the diagnostic value of CK-18 [9, 10]. Thus, the accurate information in this regard is desperately required. In this work, we performed a systematic review and meta-analysis aiming to summarize the results of published studies regarding the diagnostic effect of the serum biomarkers in NAFLD/NASH.

## 2. Methods

### 2.1. Data Sources and Searches

Articles published up to September 2016 were extracted from 4 electronic databases: PubMed, Embase, ISI Web of Science, and the Cochrane Library. Two authors (LH and LFD) independently conducted a medical literature search and screening. The following keywords combined with their corresponding MeSH terms were used: (NAFLD or NASH) and (“Serum biomarkers” or “Cytokeratin 18” or “Fibroblast growth factor 21” or “Combined biomarker”). Additionally, the references cited in retrieved articles were scrutinized by manual search. The final results were reached after discussion.

### 2.2. Inclusion Criteria

Inclusion criteria were as follows: (1) studies investigated the diagnostic value of serum biomarkers for NAFLD and contained adequate data to construct a 2 × 2 diagnostic table. (2) In each study, the final diagnosis of NAFLD had to be confirmed by histopathology of liver biopsy as the reference standard. (3) The individual study results at least were presented graphically by plotting the estimate of sensitivity and specificity of above-mentioned biomarkers for diagnosis of NAFLD. (4) There is no restriction on study design. Prospective studies, retrospective studies, and comparative studies were all considered for inclusion. (5) Only full-text articles were included for the meta-analysis.

### 2.3. Exclusion Criteria

The following studies were excluded: (1) those that were not research articles, including reviews, case reports, and letters to the editors; (2) duplication of records; (3) different articles from a primary study that contained overlapping data cohorts; and (4) the studies associated between biomarkers and NAFLD in vitro or in vivo animal experiments.

### 2.4. Quality Assessment

The methodology of selected studies was evaluated using Cochrane's Quality Assessment of Diagnostic Accuracy Studies (QUADAS) [36, 37], and the results were demonstrated by using Revman 5.3 (The Nordic Cochrane Centre, Copenhagen, Denmark) with each item scored as “yes,” “no,” or “unclear.” Two authors (LH and LFD) independently reviewed each included study. The disagreement was resolved after discussion.

### 2.5. Data Extraction

Two independent investigators (LH and LFD) carried out the data extraction by a predefined database. The disagreements of these two databases were resolved by joint reevaluation. The following key study characteristics were abstracted from each included study: the first author's name, publication year, study region, study design, numbers of cases and controls, assay method, and accompanying diagnostic thresholds (cut-offs). The number of the true positive, false-positive, false negative, and true negative results was calculated according to liver biopsy and reported cut-off for each diagnosing biomarker. A 0.5 value was automatically added to cells with 0 for adjustment [38].

### 2.6. Statistical Methods

We used Stata software, version 12.0 (Stata Corp LP, College Station, TX), and Review Manager 5.3 for the meta-analysis; data synthesis was done by using a bivariate approach. The overall diagnostic performance of serum biomarkers was evaluated by using area under the curve on summary receiver operating characteristic curves (AUROC); the combined diagnostic score and diagnostic odds ratio were also used to describe the overall diagnostic ability of the serum biomarkers. Based on 2 × 2 diagnostic tables extracted from the included studies, the diagnostic accuracy of the biomarkers was measured by combined sensitivity, specificity, positive likelihood ratio (PLR), and negative likelihood ratio (NLR). Data were presented with 95% confidence intervals (CIs) and were visualized on forest plots. For heterogeneity assessments, the Cochran *Q* method was applied and quantified with the *I*^2^ value. The definitions of heterogeneity were as follows: An *I*^2^ value less than 50% was deemed low heterogeneity, and when the *I*^2^ value was greater than 50%, substantial heterogeneity was considered [36].

## 3. Results

### 3.1. Literature Search

**Figure 1:**
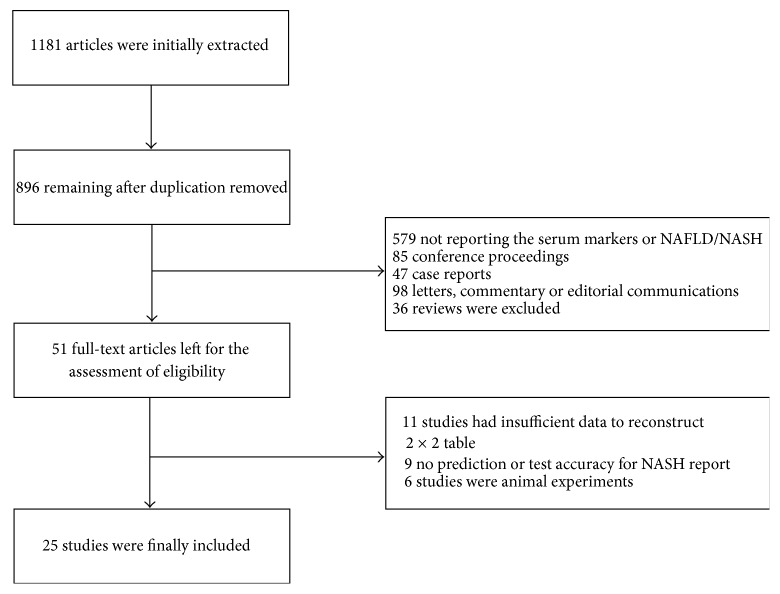
Flow diagram demonstrating literature search and selection of studies of CK-18, FGF-21, and combined biomarker panel for diagnosing NASH.

The detailed manuscript screening processes were shown in Figure 1. Briefly, 1181 articles were initially identified from above-mentioned four electronic databases. A total of 896 manuscripts that remained after duplicates were removed, among which 845 manuscripts were excluded by reviewing title and abstract for meeting the prespecified exclusion criteria. Of these manuscripts, 579 studies did not report markers or NAFLD/NASH, 85 were conference proceedings, 47 were case reports, 98 were letters, commentary or editorial communications, and 36 were reviews. After full-text assessment of eligible 11 studies contained inadequate data with which to construct a 2 × 2 diagnostic table, nine manuscripts were excluded for not reporting the prediction or accuracy of diagnosis, and six animal experiments were removed. Therefore, a total of 25 manuscripts which presented data on the NAFLD/NASH diagnostic efficiency with CK-18, FGF-21, and combined biomarker panel (CBP) finally met the inclusion criteria and were included in this meta-analysis [11–35].

### 3.2. Characteristics of Included Studies

Of the 25 included manuscripts, with the publication years spanning 2006 to 2015, 20 were published after 2010, and all 25 articles were published in English. 15 studies involved 1406 patients with data on CK-18, 12 studies involved 1943 patients with data on FGF-21, and seven studies involved 748 patients with data on CBP based on CK-18 and/or FGF-21. All of them are cross-sectional studies. Of these 25 studies, six were conducted in China, nine were conducted in United States, and 10 were from Europe. For measurement of serum biomarkers concentrations, all of included studies used blood samples that were tested by ELISA. We provide the characteristics of the 25 manuscripts in Table 1.

The overall methodology quality of the included studies was evaluated by 2 of the authors (LH and LFD) according to QUADAS [37]. All studies met the following applicability concern criteria: the included patients, setting, and reference standard match the quality assessment items; meanwhile, the index test or interpretation was brought into correspondence with the review questions mentioned in QUADAS. In addition, More than 70% of studies also met items for risk of bias criteria. In this regard, the patient selection was unclear in Musso et al. [16], and three studies [17, 24, 31] also were described as unclear in index test domain. Furthermore, the risk of bias in the reference standard domain was evaluated as high in four studies [16, 22, 27, 31], but it was unclear in Giannini et al. [34] and Yilmaz and Eren [25]. There was a high risk of bias in time and flowing domain in Feldstein et al. [14], Malik et al. [17], and Yilmaz et al. [27], but it was unclear in four studies [11, 12, 20, 22], the details of which were all summarized in Supplementary Figure S1 in Supplementary Material available online at https://doi.org/10.1155/2017/9729107.

### 3.3. Noninvasive Diagnostic Performance of Circulating Biomarkers

NASH is a potentially serious condition associated with a significant increase in overall and liver-related morbidity and mortality, because a proportion of patients with NASH develop cirrhosis and HCC [4, 5, 39]. Until now, liver biopsy remains the gold standard for diagnosing and grading NASH. However, it is obvious that this invasive procedure is not suitable as a screening test for such a prevalent condition, and this in turn restricts new promising therapies currently being tested in large clinical trials [40]. For all of these reasons, there is an urgent need to develop and validate simple, reproducible, noninvasive tests that accurately distinguish NASH from NAFLD and determine the stage and grade of the disease.

### 3.4. CK-18

CK-18 is a major intermediate filament protein in hepatocytes. Apoptosis of hepatocytes is further associated with the release of caspase-cleaved and uncleaved CK-18 fragments, namely, M30 and M65 [41]. In this study, a total of 15 studies reported concentrations of CK-18 fragments including M30 and M65 in patients who developed NASH (Table 1).

### 3.5. Meta-Analysis on Group M30

**Figure 2:**
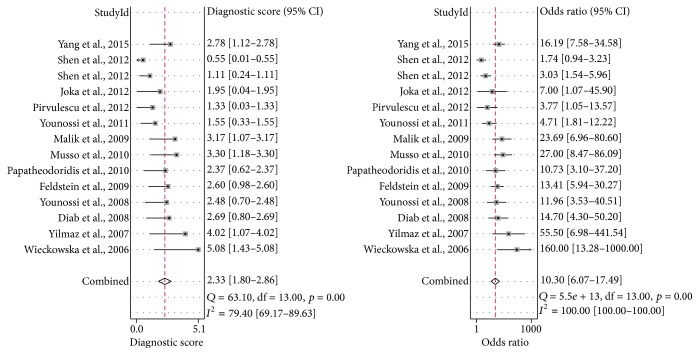
Combined DS and DOR of CK-18, M30 fragment. DS, diagnostic score. DOR, diagnostic odds ratio. CI, confidence interval.

**Figure 3:**
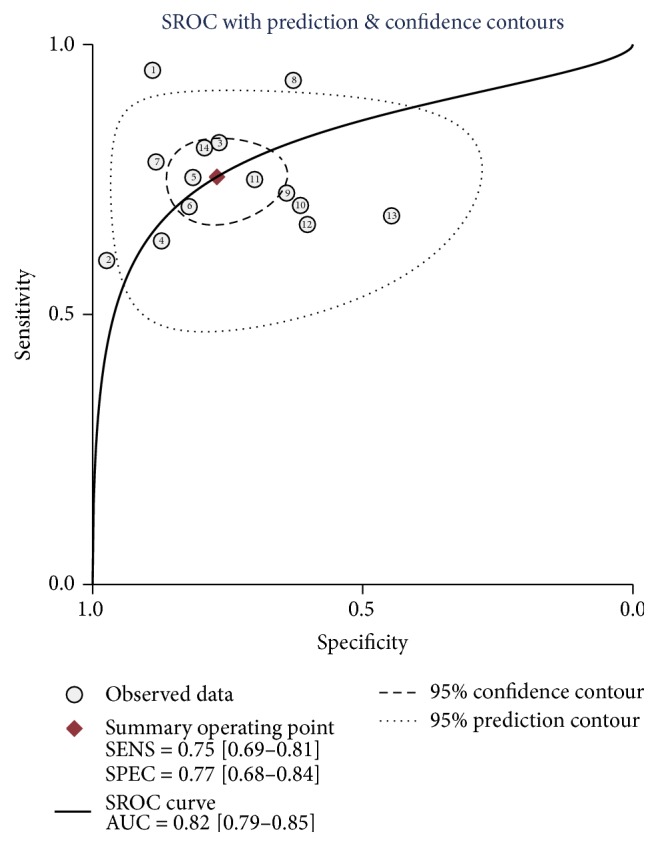
Summary receiver's operative characteristics of CK-18, M30 fragment. AUC, area under the curve. SENS, sensitivity. SPEC, specificity.

For the including studies, the cut-offs chosen ranged 121.6–380.2 U/L (0.60–0.95 sensitivity and 0.60–0.97 specificity). The AUROC for these M30 testing studies ranged 0.66–0.93 (Supplementary Table S1). The combined diagnostic score and diagnostic odds ratio were 2.33 (95% CI, 1.80–2.86) and 10.30 (95% CI, 6.07–17.49), respectively (Figure 2). The combined sensitivity, specificity, PLR, and NLR of M30 were 0.75 (95% CI, 0.69–0.81), 0.77 (95% CI, 0.68–0.84), 3.28 (95% CI, 2.32–4.65), and 0.32 (95% CI, 0.25–0.41), respectively (Supplementary Figure S2). In addition, the AUROC value of the M30 testing in predicting NASH was 0.82 (95% CI, 0.79–0.85), showing ability to distinguish NASH from NAFLD (Figure 3).

### 3.6. Meta-Analysis on Group M65

In the including 6 studies, the cut-offs ranged 243.8–790 U/L (0.62–1 sensitivity and 0.65–0.89 specificity) and the AUROC for these studies ranged 0.71–0.93 (Supplementary Table S2). According to the reported concentrations of M65, the summary diagnostic score and diagnostic odds ratio were 2.12 (95% CI, 1.51–2.72) and 8.31 (95% CI, 4.55–15.19), respectively (Supplementary Figure S3). This corresponds to a combined sensitivity, specificity, PLR, and NLR of 0.71 (95% CI, 63%–78%), 0.77 (95% CI, 0.67–0.84), 3.09 (95% CI, 2.11–4.54), and 0.37 (95% CI, 0.28–0.49), respectively (Supplementary Figure S4). As the AUROC value of M65 was 0.80 (95% CI, 0.76–0.83) (Supplementary Figure S5), this indicated that M65 and M30 testing had similar performance to distinguish NASH from simple steatosis. However, its overall diagnostic accuracy and stability need to be verified by more research data.

### 3.7. FGF-21

FGF-21, a liver-secret hormone, has recently been shown to possess beneficial effects on lipid metabolism and hepatic steatosis [42]. Several studies demonstrated that serum FGF-21 concentrations were associated with hepatic fat content especially in subjects with moderate hepatic steatosis [28, 43, 44]. In this meta-analysis, there were 12 studies reporting on the testing of FGF-21 concentrations in NAFLD patients (Table 1 and Supplementary Table S3).

### 3.8. Meta-Analysis on FGF-21

The concentrations of FGF-21 of the patient who developed NAFLD were significantly higher than control group; the summary standardized mean difference (SMD) was 1.37 (95% CI, 0.54–2.21) (Supplementary Figure S6). Particularly, in the subgroup analysis performed between simple steatosis group and NASH group, the SMD was more remarkable (SMD 1.47, 95% CI, 0.13–3.07) in NASH than the studies that tested from simple steatosis (SMD 1.12, 95% CI 0.27–1.97) (Supplementary Figure S7). These results suggest that the serum FGF-21 can be potentially used as a biomarker for NAFLD/NASH.

**Figure 4:**
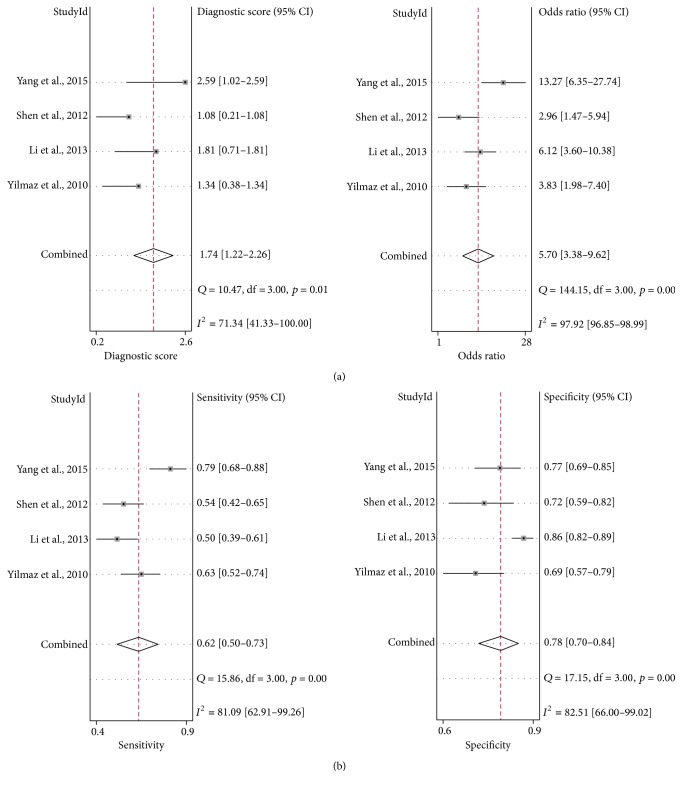
Combined DS, DOR (a), and sensitivity and specificity (b) of FGF-21. DS, diagnostic score. DOR, diagnostic odds ratio. CI, confidence interval.

Then, we further identified four studies that pooled the sensitivity and specificity of FGF-21 testing on NASH. The diagnostic score, diagnostic odds ratio, sensitivity, and specificity were 1.74 (95% CI, 1.22–2.26), 5.70 (95% CI, 3.38–9.62), 0.62 (95% CI, 0.50–0.73), and 0.78 (95% CI, 0.70–0.84), respectively (Figure 4). However, we did not extract enough data from included studies to estimate the AUROC value of FGF-21.

### 3.9. CBP

The development of NAFLD is related to inflammation, hepatocyte apoptosis, and fibrosis during disease progression [45]. Meanwhile, different noninvasion biomarkers, such as CK-18, FGF-21, and IL-1Ra, involved in NAFLD progression were significantly correlated with NAS score and the pathological characteristics of NAFLD [26]. Importantly, some reviews recently indicated that combining two or more individual biomarkers as a panel could obtain a better predictive value for NASH [7, 8, 46].

### 3.10. Meta-Analysis on CBP

The diagnostic accuracy of the biomarker panel for NASH was reported in seven studies. In these studies, as shown in Supplementary Table S4, the CBP was designed including CK-18, FGF-21, and other different markers.

**Figure 5:**
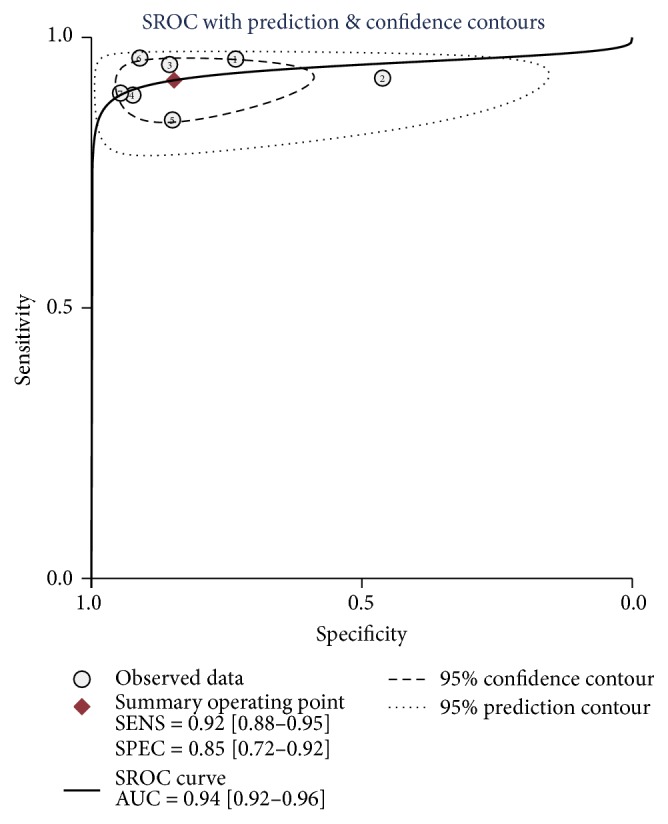
Summary receiver's operative characteristics of CBP. AUC, area under the curve. SENS, sensitivity. SPEC, specificity.

**Figure 6:**
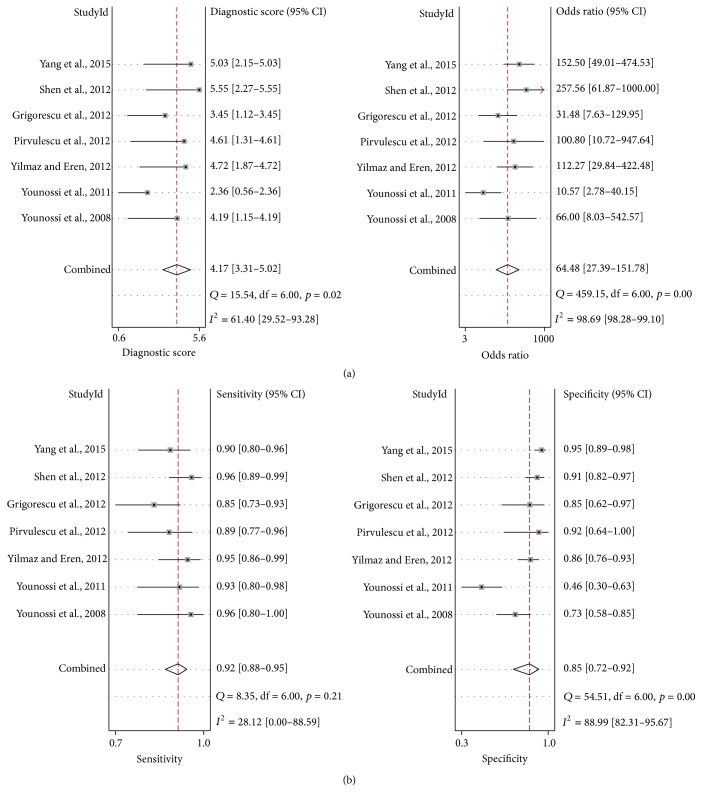
Pooled DS, DOR (a), and sensitivity and specificity (b) of CBP. DS, diagnostic score. DOR, diagnostic odds ratio. CI, confidence interval.

The AUROC value of the CBP was 0.94 (95% CI, 0.92–0.96), compared to CK-18 or FGF-21 assay, which showed the most significant ability to distinguish NASH from simple steatosis (Figure 5). The combined diagnostic score and diagnostic odds ratio of the CBP were 4.17 (95% CI, 3.31–5.02) and 64.48 (95% CI, 27.39−151.78), respectively (Figure 6(a)). The combined sensitivity and specificity were 0.92 (95% CI, 0.88–0.95) and 0.85 (95% CI, 0.72–0.92), respectively (Figure 6(b)).

### 3.11. Test of Cross-Study Heterogeneity

There was marked cross-study heterogeneity in the CK-18 fragment M30, FGF-21, and the CBP in pooled diagnostic score, sensitivity, specificity, PLR, and NLR; the *I*^2^ values were shown in Supplementary Table S5, whereas moderate heterogeneity was observed in the CK-18 fragment M65 combined diagnostic score, specificity, PLR, and NLR; the *I*^2^ values were 42.89%, 43.52%, 0.00%, and 38.13%, respectively.

## 4. Discussion

In view of the remarkable increase in prevalence of NAFLD in conjunction with the significant research effort in developing novel therapies for patients with NASH, noninvasion, convenient, reproducible, and reliable serum biomarkers are greatly needed. However, due to technical and accuracy issues, the noninvasion biomarkers for diagnosis of NAFLD, especially distinguishing NASH from NAFLD, have not been widely used all over the world.

In this systematic review, we investigated evidence for diagnostic capability of serum noninvasion biomarkers in NAFLD. Over all, the findings from the meta-analysis indicate that upregulated levels of CK-18 and FGF-21 in serum are associated with increased risk for NASH. Particularly, the diagnostic panels which combined with several biomarkers including CK-18 and/or FGF-21 showed excellent performance for distinguishing NASH from NAFLD. To our knowledge, this is the first ever meta-analysis in which diagnostic value of the most studied principal biomarkers is compared simultaneously in NAFLD patients. Despite the increasing number of studies and reviews concerning serum noninvasion biomarkers for NAFLD, there is no consensus regarding which biomarkers have best diagnostic value. Only a few systematic reviews evaluated the diagnostic capability of CK-18 based on its sensitivity and specificity [9, 10] but did not summarize and compare with other similar biomarkers.

As is well known, laboratory tests that are routinely included in the evaluation of patients with suspected NAFLD include a serum panel of liver tests (alanine aminotransferase (ALT), aspartate aminotransferase, alkaline phosphatase, and gamma-glutamyl-transpeptidase). However, Mofrad et al. [47] demonstrated that the entire histological spectrum of NAFLD can be seen in patients with normal ALT values. Moreover, Kunde et al. [48] also evaluated the diagnostic accuracy of serum ALT for NASH diagnosis, which was found to be quite poor, at merely 40%. Given the limitations of serum transaminases as noninvasive selective indicators of NAFLD, overwhelming evidence showed that the certain cytokines derived from several biochemical events including insulin resistance, oxygen stress, apoptosis, or inflammation may play more important roles in the progression of NAFLD [49]. For instance, during hepatocyte apoptosis, the fragments of CK-18 can be detectable in serum of chronic liver diseases patients by ELISA [50], and this method was tested as a promising noninvasive tool in NASH diagnosis. Serum FGF-21 was also associated with liver fat content and damage, which could be the useful circulating biomarker for predicting progression in NAFLD patients [34]. Furthermore, adipose tissue contributes to NAFLD, being a source of fatty acids and cytokines such as adiponectin, imbalance of which seems to be associated with severe NAFLD [51, 52]. The data from Wree et al. further indicated that reduced adiponectin levels may establish a proinflammatory milieu, thus increasing vulnerability to lipotoxicity, exacerbating hepatocytes injury, which promotes progression from simple steatosis to NASH and even advanced hepatic fibrosis [53].

Importantly, there is general consensus that a PLR of greater than 10 and a NLR of less than 0.05 provide reliable evidence of satisfactory diagnostic performance [54]. Based on our meta-analysis, none of the above-mentioned biomarkers fulfilled the criteria to be able to satisfactorily discriminate between patients with NASH versus NAFLD. However, the overall results suggest that CK-18 (M30) has moderate accuracy for diagnosing NASH (0.75 sensitivity, 0.77 specificity). This means 75% suspected NASH patients will be identified by CK-18 and avoid a liver biopsy, while 23% of patients who were initially diagnosed as non-NASH according to CK-18 still require a liver biopsy for further identification. Also, CK-18 (M30) with NLR of 0.32 indicates a higher risk of missing NASH. Meanwhile, CK-18 (M65) is more likely a useful biomarker for identifying NASH rather than screening, due to significant homogeneity observed in pooled specificity and PLR among the studies. Furthermore, CK-18 has some additional advantage over other biomarkers. One of the reasons is the fact that CK-18 is a major component of intermediate filaments of hepatocytes, and the circulating fragment of CK-18 can specifically reflect the degree of hepatocellular apoptosis, which is a characteristic of NASH [55].

As compared to CK-18, FGF-21 improves insulin sensitivity and insulin resistance in obesity animal models [56]. Several studies also suggest that elevated serum FGF-21 is likely due to time-dependent expression of* Fgf21* mRNA in human hepatocytes, which is more related to unsaturated fatty acids but is opposite to the patterns of insulin and glucose [43, 57, 58]. Our findings suggest that FGF-21 showed excellent performance to distinguish NASH from hepatic steatosis. With a combined specificity of 0.78, FGF-21 was good at identifying NASH. Nevertheless, its ability to confirm the diagnosis was inadequate due to the fact that the number of studies included is very few. Only four studies met the inclusive criteria, which contained adequate data to construct the diagnostic table. Also, there is some heterogeneity among the studies. These two factors weaken the conclusion. However, these results provide clues about the role of FGF-21 as a key regulator of hepatic lipid metabolism in humans and suggest that serum FGF-21 can be used as a biomarker for NASH.

The most promising application of some of these novel biomarkers for the detection of simple steatosis and NASH may be in the combination of several into diagnostic panels. In this study, we evaluated the diagnosis effect with the CBP. With the pooled AUROC of 0.94, the biomarker panel showed the most excellent diagnostic performance for diagnosis of NASH. Its pooled sensitivity and specificity were 0.92 and 0.85 in contrast to those of CK-18 or FGF-21, which had the highest sensitivity and specificity. Therefore, during noninvasive diagnosis and monitoring NASH procedure, when CK-18 or FGF-21 initially diagnoses NASH, additional steps with other biomarkers should be performed in case of false-positive results.

The remarkable cross-study heterogeneity was found in this meta-analysis, which may have been due to factors like methodology quality, country, study design, and sample size. In general, the heterogeneity was more evident in the results with the circulating level of noninvasive biomarker measured by the *I*^2^ value. In the absence of a standardized operation introduction, features like the storage conditions of blood sample, antibody titer for different biomarkers, and instrument accuracy are likely to be the source of heterogeneity in included studies.

There were several limitations in this study, the most important of which was derived from the different methodologies. First, few biomarkers met the rigorous criteria for diagnostic test accuracy, suggesting that if such diagnostic test accuracy assessments were available, the present conclusions could be markedly affected. Furthermore, although the results of this meta-analysis showed that combined biomarker panel performed well for NASH diagnostic, the high *I*^2^ value indicated marked heterogeneity in included studies; a subgroup analysis according to the degree of NASH could not be conducted owing to inadequate data. With the emergence of numerous new studies about CBP, we will update the meta-analysis in further study.

## 5. Conclusion

As the first meta-analysis to comprehensively and quantitatively evaluate the relationship between noninvasive biomarkers and NAFLD, this study demonstrates that CK-18, FGF-21, and related biomarker panel can be used to diagnose NAFLD, especially NASH. Importantly, use of CBP resulted in improved accuracy with highest sensitivity and specificity, when compared to use of single biomarker including CK-18 and FGF-21. Further research is required to validate** t**he most optimized pooled biomarker panel in a well-structured, population-based cohort study with blinded evaluation. Additionally, understanding how above-mentioned biomarkers influence NASH progression may help to elucidate potentially biological mechanisms for determining treatment strategies and prognosis.

## Supplementary Material

The methodology quality assessment of included studies was shown in Figure S1. Figure S2–S5 indicated the results of analysis on CK-18 reporting sensitivity, specificity and summary ROC curves. Standardised mean differences in FGF-21 concentration between subgroups were shown in Figure S6 and S7. Table S1–S4 demonstrated the study characteristics and test results of included studies, grouped by CK-18, FGF-21 and combined biomarker panel. Table S5 showed the cross-study heterogeneity of different biomarkers.

## Figures and Tables

**Table 1:** Major characteristics of the studies included in the meta-analysis.

Number	Study (year)	Country	Study design	Number of cases	Age, y (SD)	Gender (M/F)	Methods	Biomarkers
1	Wieckowska et al. [11] (2006)	USA	Cross-sectional	39	50.8 (11.1)	18/21	ELISA	CK-18 (M30)
2	Yilmaz et al. [12] (2007)	Turkey	Cross-sectional	83	48.9 (9.1)	45/38	ELISA	CK-18 (M30 & M65)
3	Diab et al. [13] (2008)	USA	Cross-sectional	86	48.0 (11.1)	38/48	ELISA	CK-18 (M30)
4	Feldstein et al. [14] (2009)	USA	Cross-sectional	139	48.0 (1.9)	51/88	ELISA	CK-18 (M30)
5	Papatheodridis et al. [15] (2010)	Greece	Cross-sectional	58	48.0 (13.0)	32/17	ELISA	CK-18 (M30)
6	Musso et al. [16] (2010)	Italy	Cross-sectional	125	46.0 (4.0)	89/36	ELISA	CK-18 (M30)
7	Malik et al. [17] (2009)	USA	Cross-sectional	95	48.0 (5.3)	58/37	ELISA	CK-18-M30
8	Shen et al. [18] (2012)	China	Cross-sectional	147	47.7 (9.7)	121/99	ELISA	CK-18 (M30 & M65)
9	Joka et al. [19] (2012)	Germany	Cross-sectional	22	45.6 (3.3)	15/7	ELISA	CK-18 (M30 & M65)
10	Younossi et al. [20] (2008)	USA	Cross-sectional	69	41.6 (10.6)	23/46	ELISA	Combined biomarker panel
11	Younossi et al. [21] (2011)	USA	Cross-sectional	79	42.3 (10.3)	18/61	ELISA	Combined biomarker panel
12	Pirvulescu et al. [22] (2012)	Romania	Cross-sectional	60	44.9 (9.4)	18/42	ELISA	Combined biomarker panel
13	Grigorescu et al. [23] (2012)	Romania	Cross-sectional	79	43.7 (11.1)	56/23	ELISA	Combined biomarker panel
14	Shen et al. [24] (2012)	China	Cross-sectional	220	48.1 (9.7)	121/99	ELISA	Combined biomarker panel
15	Yilmaz and Eren [25] (2012)	Turkey	Cross-sectional	136	48.8 (7.5)	70/66	ELISA	Combined biomarker panel
16	Yang et al. [26] (2015)	China	Cross-sectional	270	30.3 (12.7)	128/142	ELISA	Combined biomarker panel
17	Yilmaz et al. [27] (2010)	Turkey	Cross-sectional	159	47.0 (8.0)	71/88	ELISA	FGF-21
18	Li et al. [28] (2010)	China	Cross-sectional	348	43.5 (11.1)	212/136	ELISA	FGF-21
19	Dushay et al. [29] (2010)	Spain	Cross-sectional	21	31 (10.0)	7/14	ELISA	FGF-21
20	Dasarathy et al. [30] (2011)	USA	Cross-sectional	26	43.3 (7.0)	13/13	ELISA	FGF-21
21	Reinehr et al. [31] (2012)	USA	Cross-sectional	60	12.0 (1.4)	30/30	ELISA	FGF-21
22	Li et al. [32] (2013)	China	Cross-sectional	712	51.4 (12.9)	278/434	ELISA	FGF-21
23	Shen Y et al. [33] (2013)	China	Cross-sectional	74	63.9 (8.6)	39/35	ELISA	FGF-21
24	Giannini et al. [34] (2013)	USA	Cross-sectional	217	15.0 (0.4)	91/126	ELISA	FGF-21
25	Alisi et al. [35] (2013)	Italy	Cross-sectional	107	10.5 (4.8)	41/66	ELISA	FGF-21

SD, standard deviation. M, male. F, female.
