# Supplementary material for: Diagnostic Value of CK-18, FGF-21, and Related Biomarker Panel in Nonalcoholic Fatty Liver Disease: A Systematic Review and Meta-Analysis
Source: Biomed Res Int. 2017 Feb 23;2017:9729107. doi: 10.1155/2017/9729107 (PMC5343245; doi:10.1155/2017/9729107)
Supplement: Supplementary file 1 — The methodology quality assessment of included studies was shown in Figure S1. Figure S2–S5 indicated the results of analysis on CK-18 reporting sensitivity, specificity and summary ROC curves. Standardised mean differences in FGF-21 concentration between subgroups were shown in Figure S6 and S7. Table S1–S4 demonstrated the study characteristics and test results of included studies, grouped by CK-18, FGF-21 and combined biomarker panel. Table S5 showed the cross-study heterogeneity of different biomarkers. [file 9729107.f1.pdf]

Supplementary Material is composed of Figure S1-S7 and Table S1-S5, all of them may be found in the online version of this article.

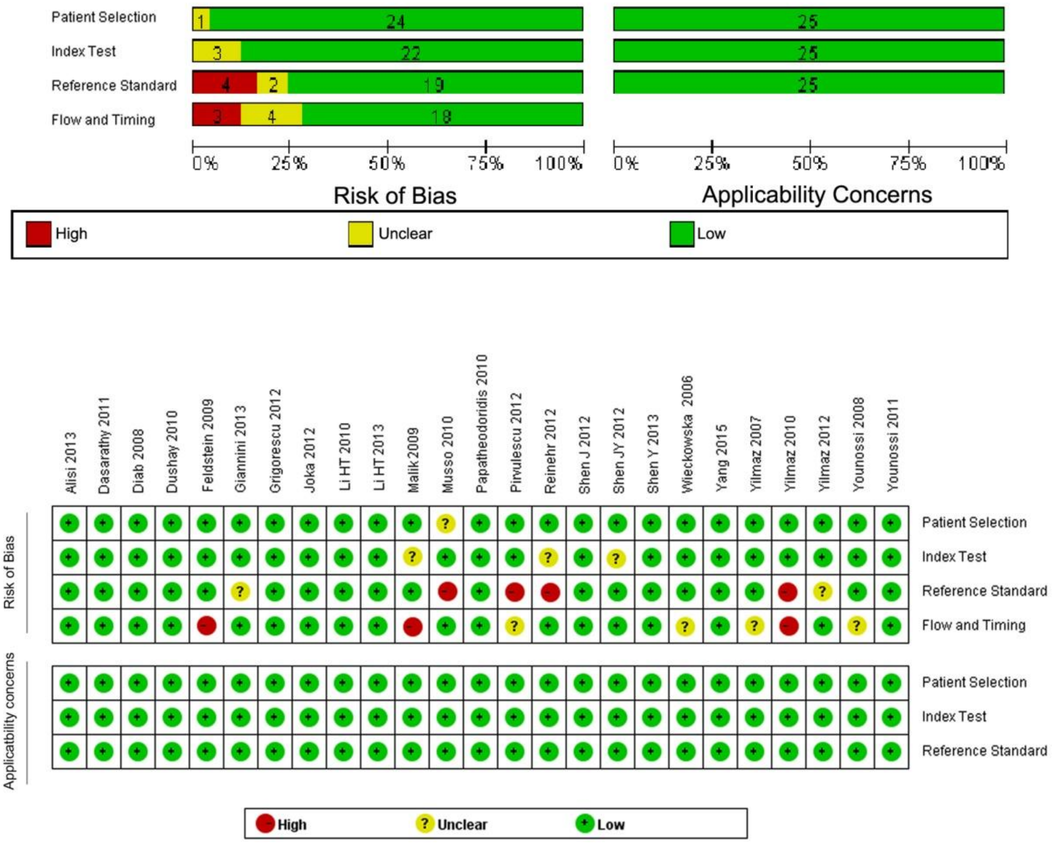

Figure S1 Methodology quality evaluated according to Quality Assessment of Studies of Diagnostic Accuracy (QUADAS-2).

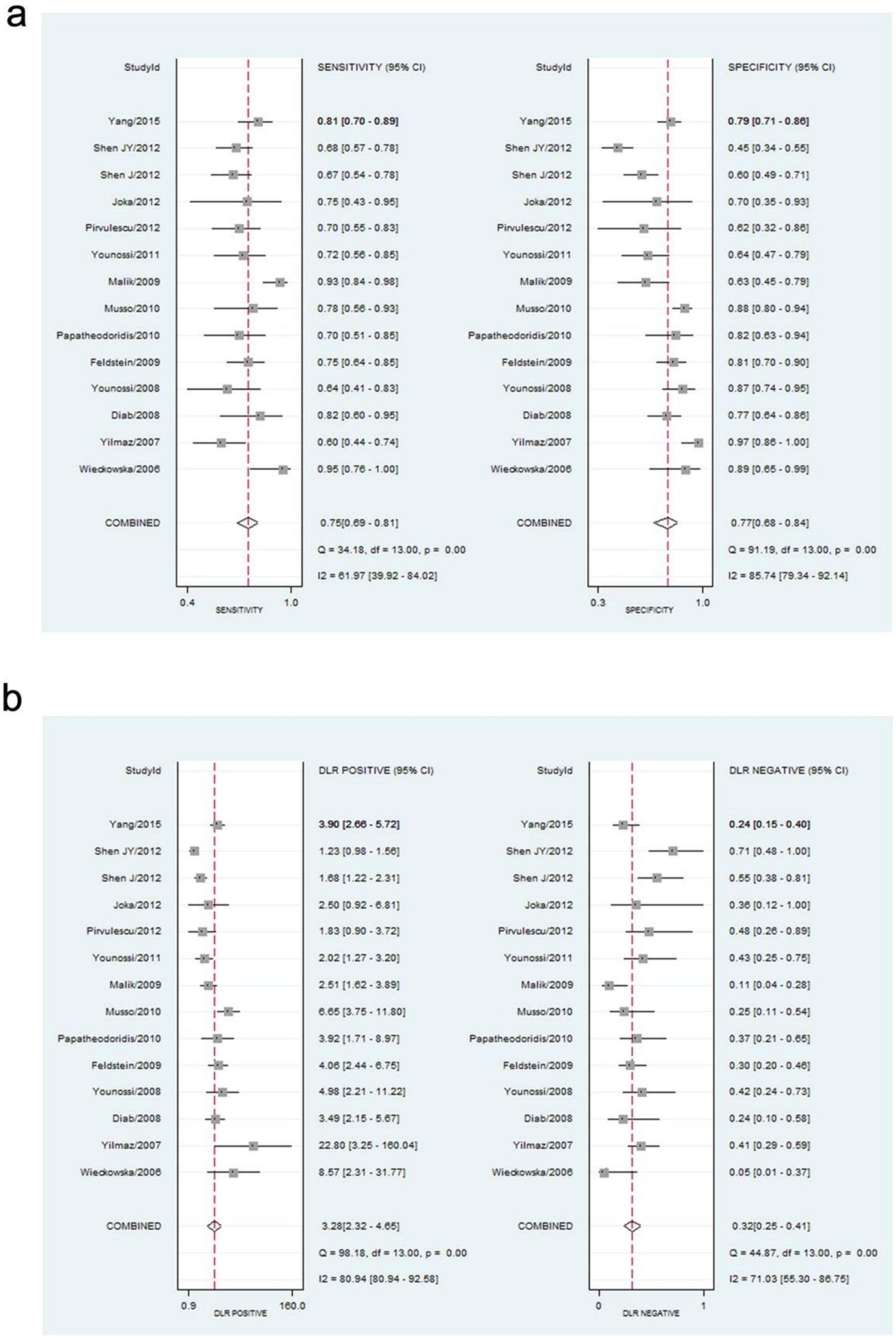

Figure S2 Combined sensitivity, specificity (**a**) and DLR positive, DLR negative (**b**) of CK-18, M30 fragment.

DLR, diagnostic likelihood ratio; CI, confidence interval.

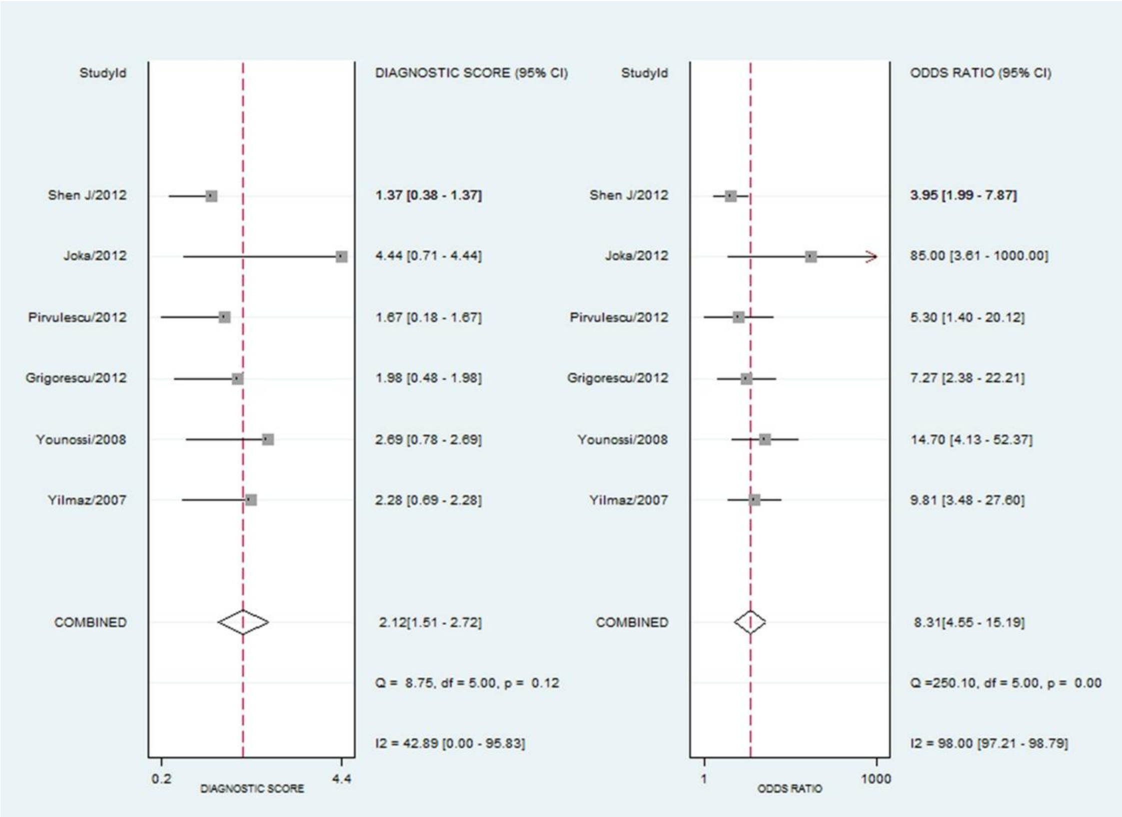

Figure S3 Combined DS and DOR of CK-18, M65 fragment.

DS, diagnostic score. DOR, diagnostic odds ratio. CI, confidence interval.

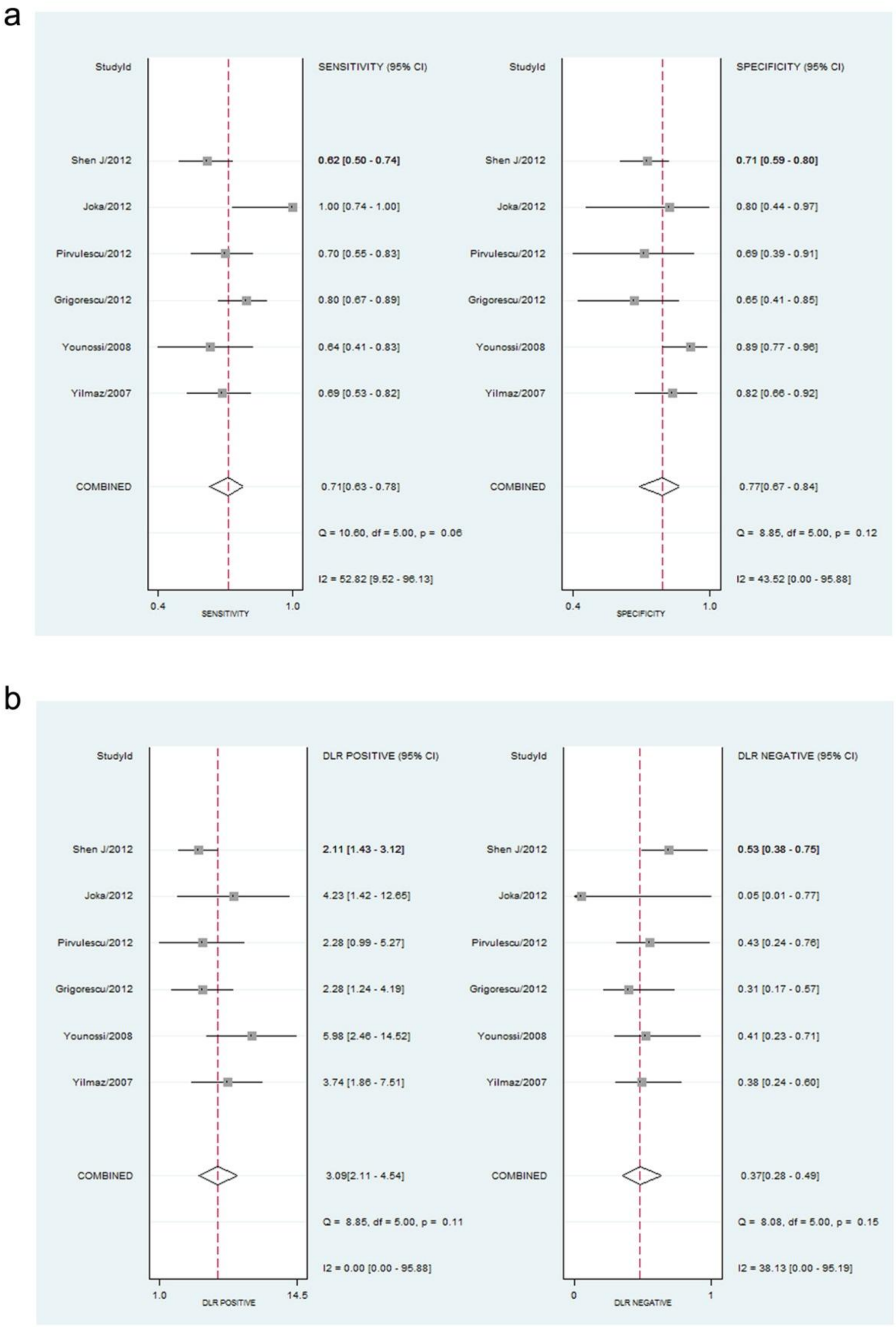

Figure S4 Combined sensitivity, specificity (**a**) and DLR positive, DLR negative (**b**) of CK-18, M65 fragment.

DLR, diagnostic likelihood ratio; CI, confidence interval.

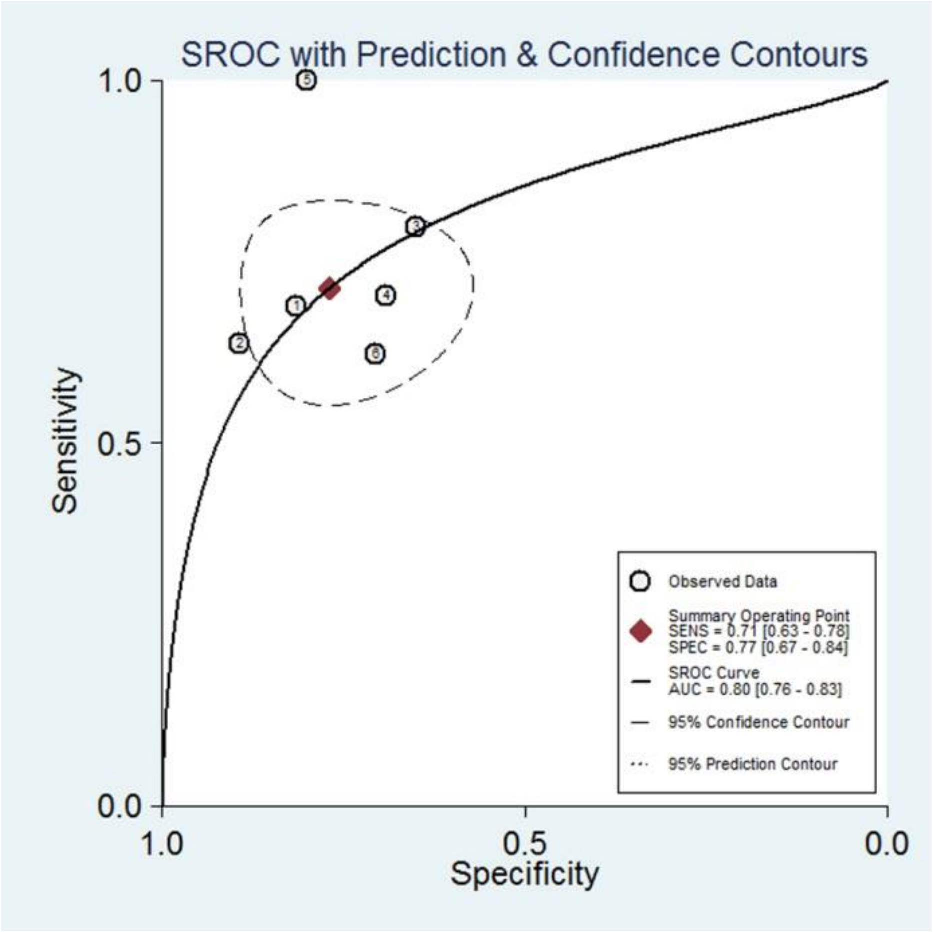

Figure S5 Summary receiver's operative characteristics (SROC) of CK-18, M65 fragment. AUC, area under the curve. SENS, sensitivity. SPEC, specificity.

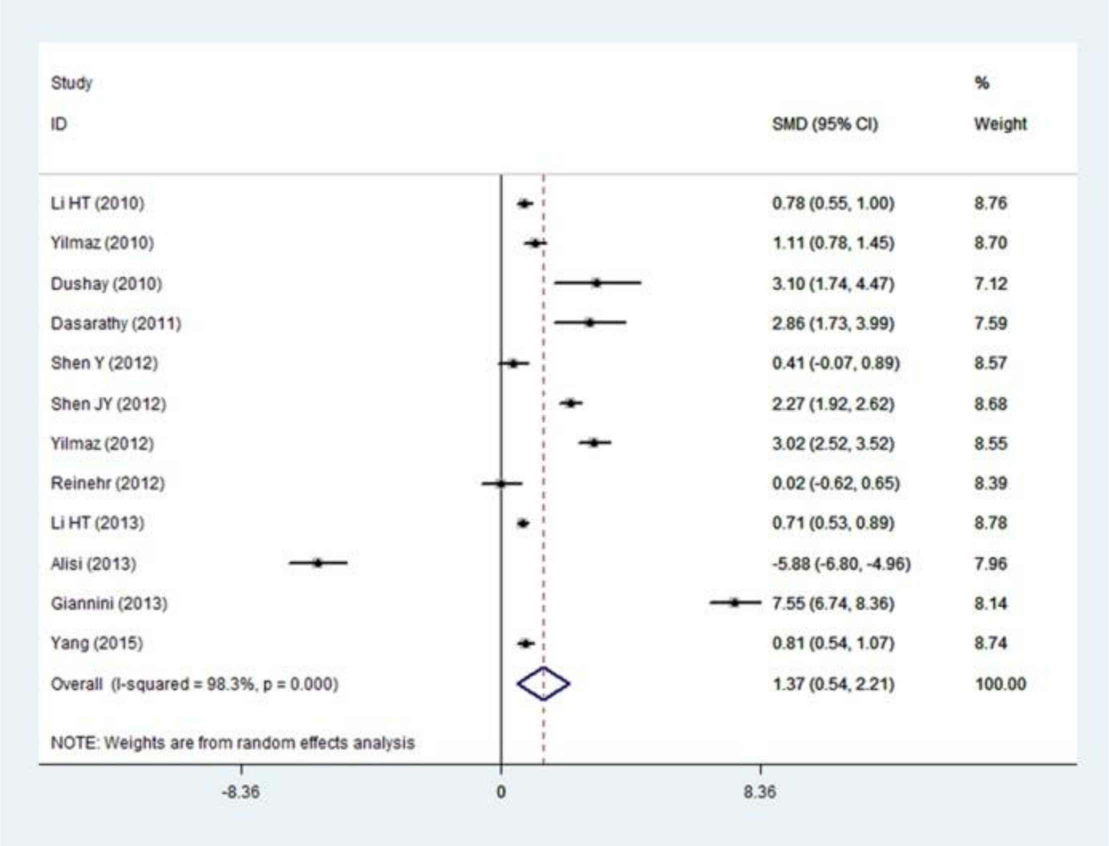

Figure S6 Forest plots showing SMD in FGF-21 concentration between the patients with NAFLD (simple steatosis and NASH) and normal people in all studies reporting data as continuous variables.

Negative values indicate lower concentrations. Positive values indicate higher concentrations prior to NAFLD. SMD, standardised mean differences.

a

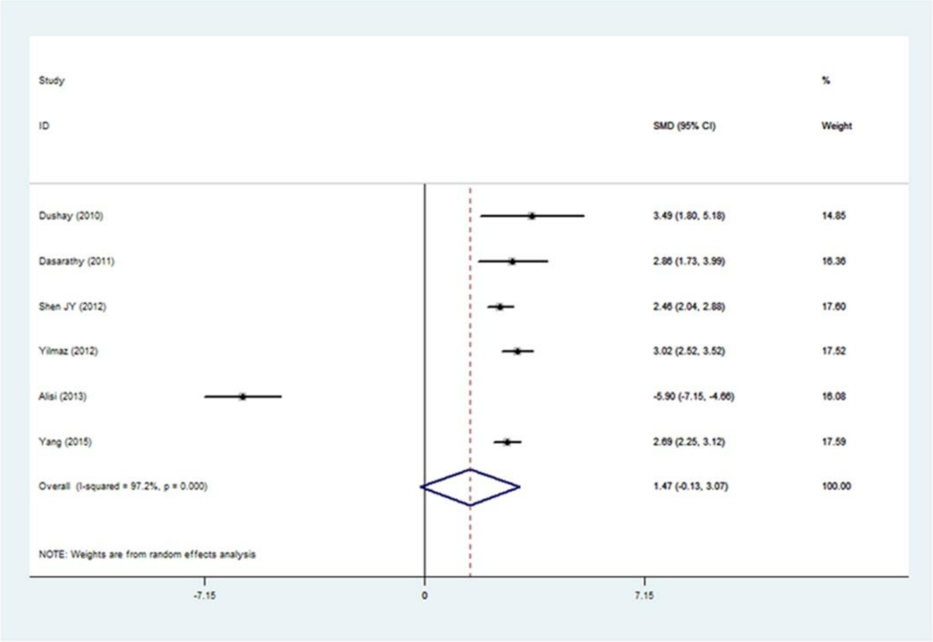

b

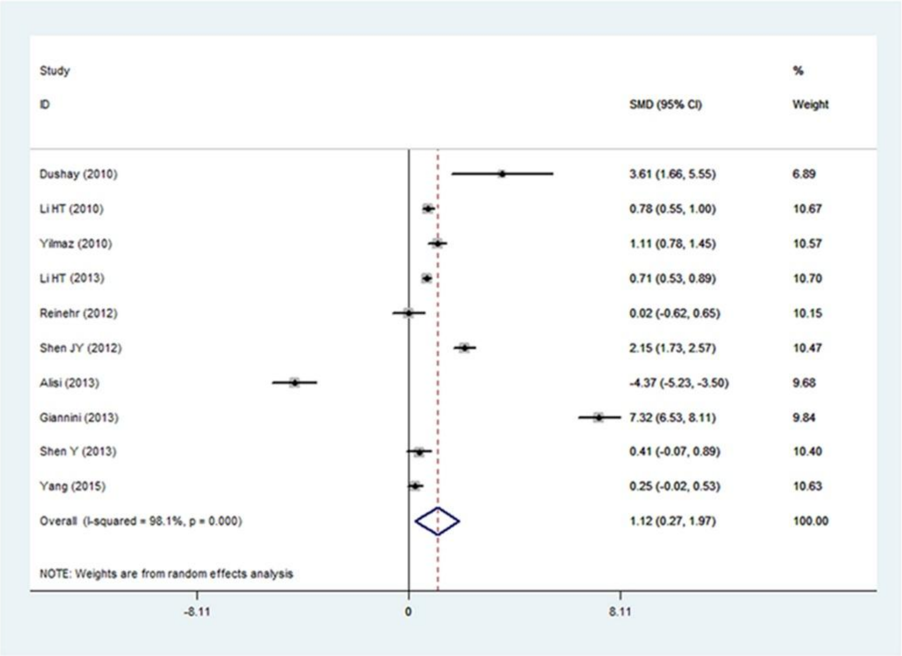

Figure S7 SMD of FGF-21 concentration in subgroups including NASH *vs.* normal control (a) and simple steatosis *vs.* normal control (b).

Negative values indicate lower concentrations. Positive values indicate higher concentrations prior to NASH or simple steatosis. SMD, standardised mean differences.

Table S1 Overview of 14 studies on CK-18 fragment M30 reporting the results in accuracy of diagnosis

Study (y)	Cut-off	Sensitivity (95% CI)	Specificity (95% CI)	PLR (95% CI)	NLR (95% CI)	AUROC (95% CI)
Wieckowska et al [11] (2006)	380.2 U/L	0.95 (0.76-1.00)	0.89 (0.65-0.99)	8.57 (2.31-31.77)	0.05 (0.01-0.37)	0.93 (NA)
Yilmaz et al [12] (2007)	121.6 U/L	0.60 (0.44-0.74)	0.97 (0.86-1.00)	22.80 (3.25-160.04)	0.41 (0.29-0.59)	0.73 (0.62-0.82)
Diab et al [13] (2008)	252 U/L	0.82 (0.60-0.95)	0.77 (0.64-0.86)	3.49 (2.15-5.67)	0.24 (0.10-0.58)	0.88 (0.77-0.99)
Feldstein et al [14] (2009)	246 U/L	0.75 (0.64-0.85)	0.81 (0.70-0.90)	4.06 (2.44-6.75)	0.30 (0.20-0.46)	0.83 (0.75-0.91)
Papatheodridis et al [15] (2010)	225 U/L	0.70 (0.51-0.85)	0.82 (0.63-0.94)	3.92 (1.71-8.97)	0.37 (0.21-0.65)	0.87 (0.79-0.96)
Musso et al [16] (2010)	246 U/L	0.78 (0.56-0.93)	0.88 (0.80-0.94)	6.65 (3.75-11.80)	0.25 (0.11-0.54)	0.83 (0.80-0.90)
Malik et al [17] (2009)	300 U/L	0.93 (0.84-0.98)	0.63 (0.45-0.79)	2.51 (1.62-3.89)	0.11 (0.04-0.28)	0.80 (0.76-0.84)
Shen J et al [18] (2012)	338 U/L	0.67 (0.54-0.78)	0.60 (0.49-0.71)	1.68 (1.22-2.31)	0.55 (0.38-0.81)	0.66 (0.57-0.75)
Joka et al [19] (2012)	149.5 U/L	0.75 (0.43-0.95)	0.70 (0.35-0.93)	2.50 (0.92-6.81)	0.36 (0.12-1.00)	0.77 (0.57-0.97)
Younossi et al [20] (2008)	174 U/L	0.64 (0.41-0.83)	0.87 (0.74-0.95)	4.98 (2.21-11.22)	0.42 (0.24-0.73)	0.71 (0.59-0.81)
Younossi et al [21] (2011)	273 U/L	0.72 (0.56-0.85)	0.64 (0.47-0.79)	2.02 (1.27-3.20)	0.43 (0.25-0.75)	0.71 (0.60-0,81)
Pirvulescu et al [22] (2012)	136 U/L	0.70 (0.55-0.83)	0.62 (0.32-0.86)	1.83 (0.90-3.72)	0.48 (0.26-0.89)	0.77 (0.61-0.92)
Shen JY et al [24] (2012)	338 U/L	0.68 (0.57-0.78)	0.45 (0.34-0.55)	1.23 (0.98-1.56)	0.71 (0.48-1.00)	0.70 (0.61-0.78)
Yang et al [26] (2015)	17.75 ng/L	0.81 (0.70-0.89)	0.79 (0.71-0.86)	3.90 (2.66-5.72)	0.24 (0.15-0.40)	0.86 (NA)

PLR, positive likelihood ratio. NLR, negative likelihood ratio. CI, confidence interval. NA, not available.

Table S2 Overview of six studies on CK-18 fragment M65 reporting the results in accuracy of diagnosis

Study (y)	Cut-off (U/L)	Sensitivity (95% CI)	Specificity (95% CI)	PLR (95% CI)	NLR (95% CI)	AUROC (95% CI)
Yilmaz et al [12] (2007)	243.8 U/L	0.69 (0.53-0.82)	0.82 (0.66-0.92)	3.74 (1.86-7.51)	0.38 (0.24-0.60)	0.74 (0.64-0.83)
Shen J et al [18] (2012)	790 U/L	0.62 (0.50-0.74)	0.71 (0.59-0.80)	2.11 (1.43-3.12)	0.53 (0.38-0.75)	0.71 (0.62-0.79)
Joka et al [19] (2012)	386 U/L	1.00 (0.74-1.00)	0.80 (0.44-0.97)	4.23 (1.42-12.65)	0.05 (0.01-0.77)	0.93 (0.81-1.0)
Younossi et al [20] (2008)	384 U/L	0.64 (0.41-0.83)	0.89 (0.77-0.96)	5.98 (2.46-14.52)	0.41 (0.23-0.71)	0.81 (0.70-0.90)
Pirvulescu et al [22] (2012)	389 U/L	0.70 (0.55-0.83)	0.69 (0.39-0.91)	2.28 (0.99-5.27)	0.43 (0.24-0.76)	0.82 (0.71-0.94)
Grigorescu et al [23] (2012)	340 U/L	0.80 (0.67-0.89)	0.65 (0.41-0.85)	2.28 (1.24-4.19)	0.31 (0.17-0.57)	0.79 (0.69-0.87)

PLR, positive likelihood ratio. NLR, negative likelihood ratio. CI, confidence interval.

Table S3 Overview of 12 studies on FGF-21 reporting the results in means and standard deviations.

Study (year)	NAFLD						Control		
	Simple steatosis			NASH					
	N	Mean (pg/ml)	SD	N	Mean (pg/ml)	SD	N	Mean (pg/ml)	SD
Dushay et al [29] (2010)	6	770	290	9	250	80	6	30	10
Li HT et al [28] (2010)	224	402.38	287.7	NA	NA	NA	124	198.62	205.7
Yilmaz et al [27] (2010)	82	200	108	NA	NA	NA	77	93	81.5
Dasarathy et al [30] (2011)	NA	NA	NA	10	189.7	36.3	16	114.8	17.6
Li HT et al [32] (2013)	159	361.27	261	NA	NA	NA	553	226.08	163.7
Reinehr et al [31] (2012)	12	198	173	NA	NA	NA	48	195	182
Shen JY et al [24] (2012)	64	248	67	82	334	112	74	104	67.4
Yilmaz and Eren [25] (2012)	NA	NA	NA	60	228.5	50	76	83	47
Alisi et al [35] (2013)	52	89	18.6	32	54	12.8	23	196	34.1
Giannini et al [34] (2013)	186	206	14.5	NA	NA	NA	31	99	12
Shen Y et al [33] (2013)	27	321.5	178.1	NA	NA	NA	47	256.1	149.1
Yang et al [26] (2015)	111	21.4	17.1	68	56.8	15.6	91	16.6	13.9

SD, standard deviations. NA, not available.

Table S4 Overview of seven studies on CBP reporting the results in accuracy of diagnosis

Study (y)	Markers	Sensitivity (95% CI)	Specificity (95% CI)	PLR (95% CI)	NLR (95% CI)	AUROC (95% CI)
Younossi et al [20] (2008)	CK-18 Adiponectin Resistin	0.96 (0.80-1.00)	0.73 (0.58-0.85)	3.60 (2.20-5.88)	0.05 (0.01-0.3)	0.91 (0.81-0.96)
Younossi et al [21] (2011)	CK-18 BMI Triglycerides	0.93 (0.80-0.98)	0.46 (0.30-0.63)	1.72 (1.27-2.33)	0.16 (0.05-0.51)	0.81 (0.70-0.89)
Shen JY et al [24] (2012)	CK-18 FGF21 AFABP	0.96 (0.89-0.99)	0.91 (0.82-0.97)	10.74 (5.00-23.08)	0.04 (0.01-0.13)	0.71 (0.63-0.79)
Grigorescu et al [23] (2012)	CK-18 IL-6 Adiponectin	0.85 (0.73-0.93)	0.85 (0.62-0.97)	5.65 (1.98-16.13)	0.18 (0.10-0.34)	0.90 (0.82-0.96)
Yilmaz and Eren [25] (2012)	CK-18 FGF-21 Osteoprotegerin	0.95 (0.86-0.99)	0.86 (0.76-0.93)	6.56 (3.79-11.37)	0.06 (0.02-0.18)	NA
Pirvulescu et al [22] (2012)	CK-18, BMI, ALT, AST and HOMA-IR	0.89 (0.77-0.96)	0.92 (0.64-1.00)	11.62 (1.76-76.56)	0.12 (0.05-0.27)	0.96
Yang et al [26] (2015)	CK-18, FGF-21, IL-1Ra, PEDF and OPG	0.90 (0.80-0.96)	0.95 (0.89-0.98)	16.60 (7.59-36.29)	0.11 (0.05-0.22)	NA

PLR, positive likelihood ratio. NLR, negative likelihood ratio. CI, confidence interval. NA, not available.

Table S5 Overview of cross-study heterogeneity evaluated by I^2 analysis

Biomarker		I^2				
		**Diagnostic score**	**Sensitivity**	**Specificity**	**PLR**	**NLR**
CK-18	M30	79.40%	61.79%	85.74%	80.94%	71.03%
	M65	42.89%	52.82%	43.52%	0.00%	38.13%
FGF-21		74.34%	81.09%	82.51%	39.10%	76.62%
CBP		61.40%	28.12%	88.99%	86.08%	25.98%

PLR, positive likelihood ratio. NLR, negative likelihood ratio
